# Supplementary material for: On the effectiveness of communication strategies as non-pharmaceutical interventions to tackle epidemics
Source: PLoS One. 2021 Oct 29;16(10):e0257995. doi: 10.1371/journal.pone.0257995 (PMC8555801; doi:10.1371/journal.pone.0257995)
Supplement: S1 Eq — Equations that describe the evolution in time of the states of the system, where S are susceptibles, E exposed, I infected, R removed and D dead agents. The parameters of the system are shown and explained in S1 Table. This model was proposed by [36]. (PDF) [file pone.0257995.s001.pdf]

On the effectiveness of communication strategies as non-pharmaceutical interventions to tackle epidemics

Alejandro Bernardin, Alejandro J. Martínez, Tomas Perez-Acle

Equations SEIRD model

$$\begin{aligned}
\frac{dS}{dt} &= \frac{-\beta_I SI}{N} - \frac{\beta_D SD}{N}, \\
\frac{dE}{dt} &= \frac{\beta_I SI}{N} + \frac{\beta_D SD}{N} - \frac{E}{T_E}, \\
\frac{dI}{dt} &= \frac{E}{T_E} - \frac{I}{T_I}, \\
\frac{dR}{dt} &= (1-f)\frac{I}{T_I}, \\
\frac{dD}{dt} &= f\frac{I}{T_I} - \frac{D}{T_D}.
\end{aligned}$$
