# Supplementary figures and images for: On the effectiveness of communication strategies as non-pharmaceutical interventions to tackle epidemics
Source: PLoS One. 2021 Oct 29;16(10):e0257995. doi: 10.1371/journal.pone.0257995 (PMC8555801; doi:10.1371/journal.pone.0257995)

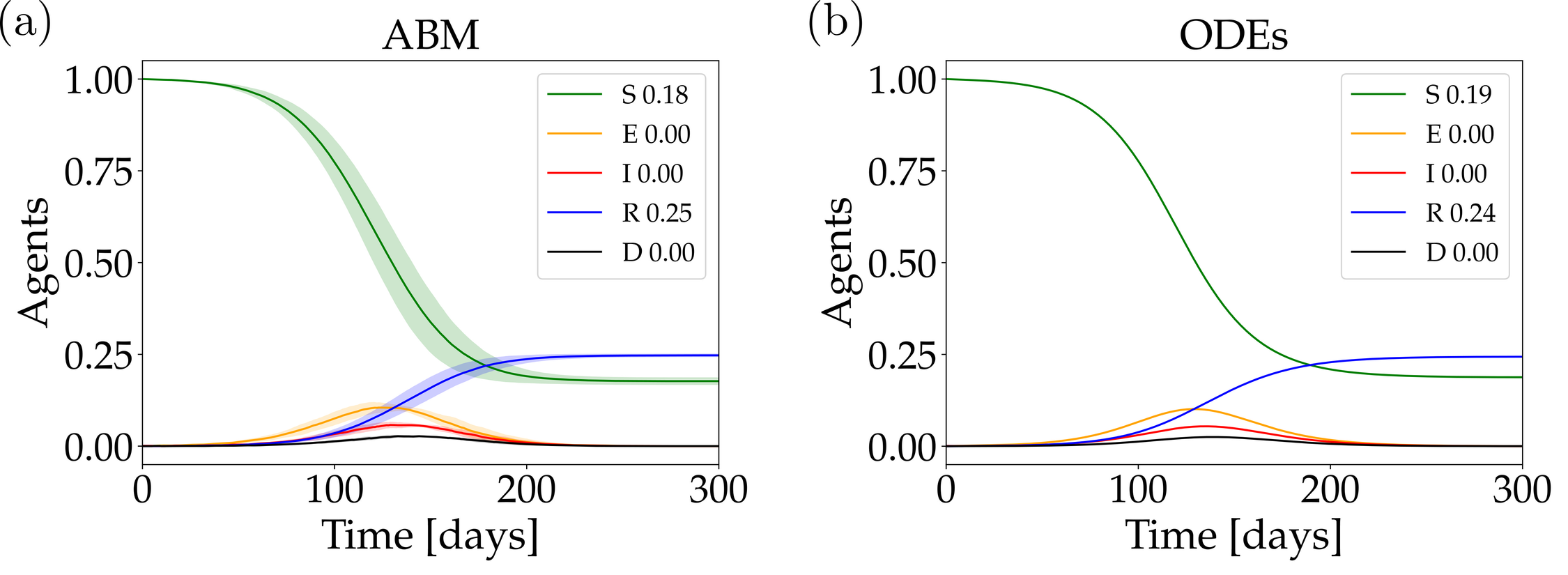

Supplement: S1 Fig — (a) ABM, temporal evolution of the states of the system for 100 repetitions. The shaded area is the standard deviation. (b) Same model in deterministic ODEs system. In both panel, curves represent the temporal evolution of the states of the system. The legend for each states represent the final ratio of agents at the end of the simulation. Maximum infected in ABM, 577 persons in t = 129 [days], maximum infected in ODE, 542 persons, in t = 135 [days]. It is worth noting that these systems do not include the information model”. (TIF) [file pone.0257995.s002.tif]

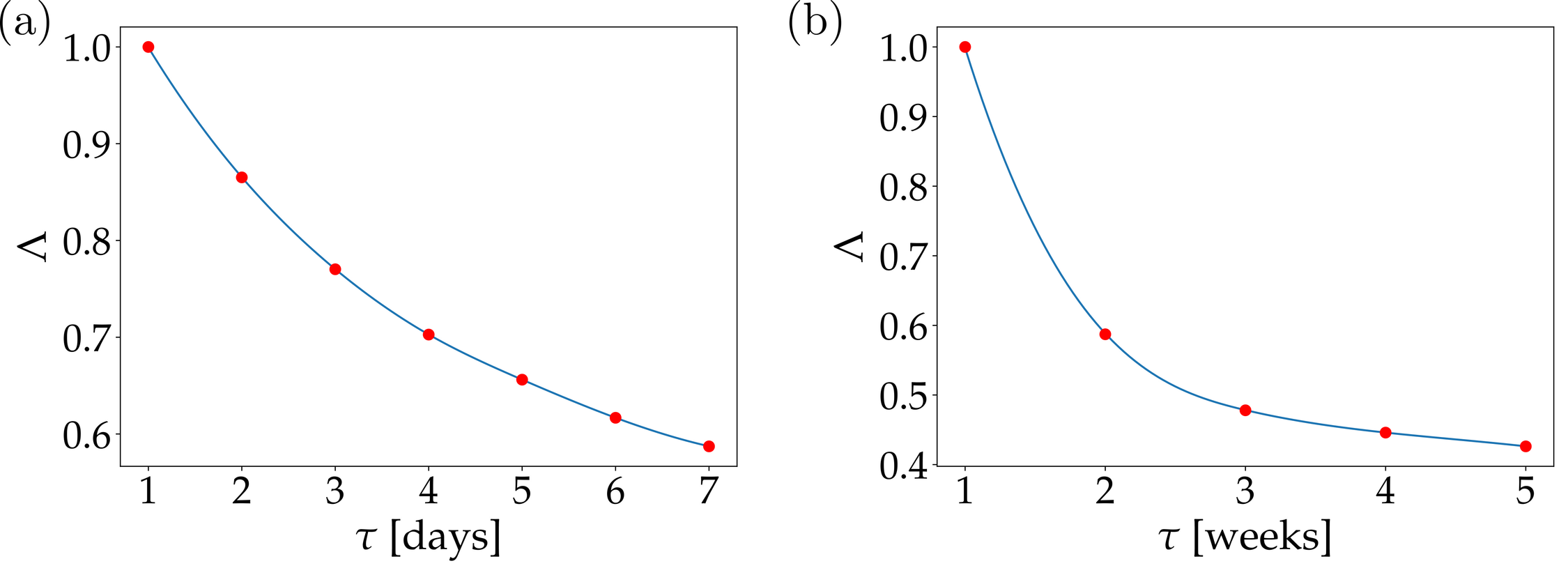

Supplement: S2 Fig — Λ represent the area under the curve for each curve τ in Fig 3b. We can see how Λ, represented by red dots, decrease as τ increase. (a) Daily analysis. (b) Weekly analysis. Continuous lines represent the tendency of the curve. (TIF) [file pone.0257995.s003.tif]

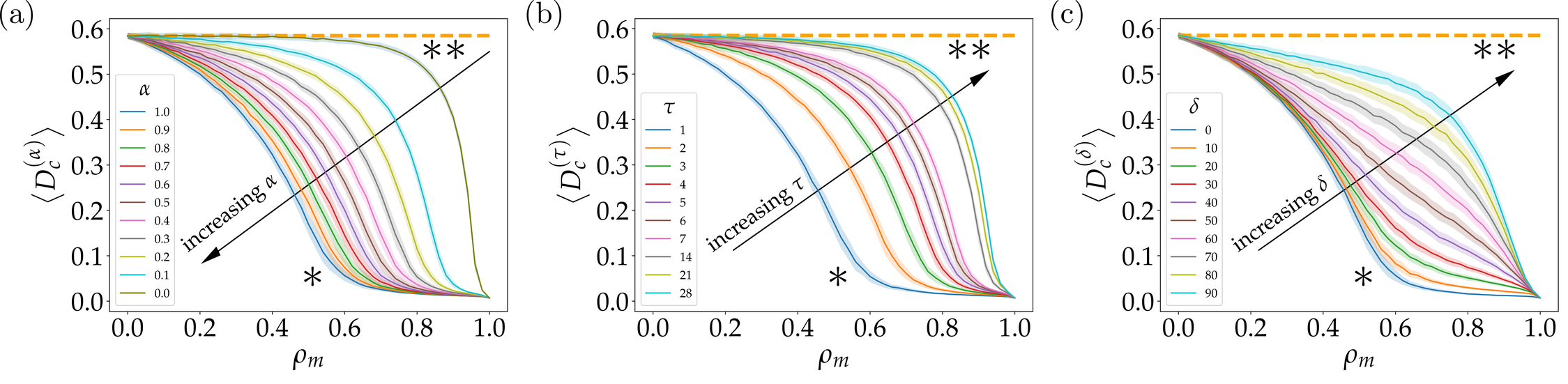

Supplement: S3 Fig — We replicate the analysis in Fig 3 but instead of susceptible agents now we have the cumulative dead agents. All panels show the final ratio of cumulative dead agents after 1000 days of simulation for different values of ρm. (a) Central information delivered to the population while the ratio of informed agents changes. Different curves represent different ratios of informed agents α, ranging from 0 (olive line) to 1 (blue line) with 0.1 interval. (b) Periodicity of information delivery. Different curves represent different periodicity of information τ, delivered to the population. We have tested periodicity daily from 1 day (blue line) to 7 days (pink line), and then weekly, at 14, 21 and 28 days (green line). (c) Delay in starting information delivery. Different curves represent different delays δ, considering the elapsed time to deliver the message from the beginning of the epidemic. We have tested delay in the first message from 0 (blue line) to 90 days (light blue line) with 10 days interval. After the first message arrives, subsequent information is delivered daily. In all panels, shaded areas represent the standard deviation for 100 simulations. A * under blue line indicates the ideal strategy, and ** below orange dashed line indicates the worst strategy, i.e., when there is no central information delivered to the population neither information delivered to infected agents. Curves with lower values of 〈Dc(x)〉 where x ∈ {α, τ, δ} implied a better strategy result, i.e., less dead agents. (TIF) [file pone.0257995.s004.tif]

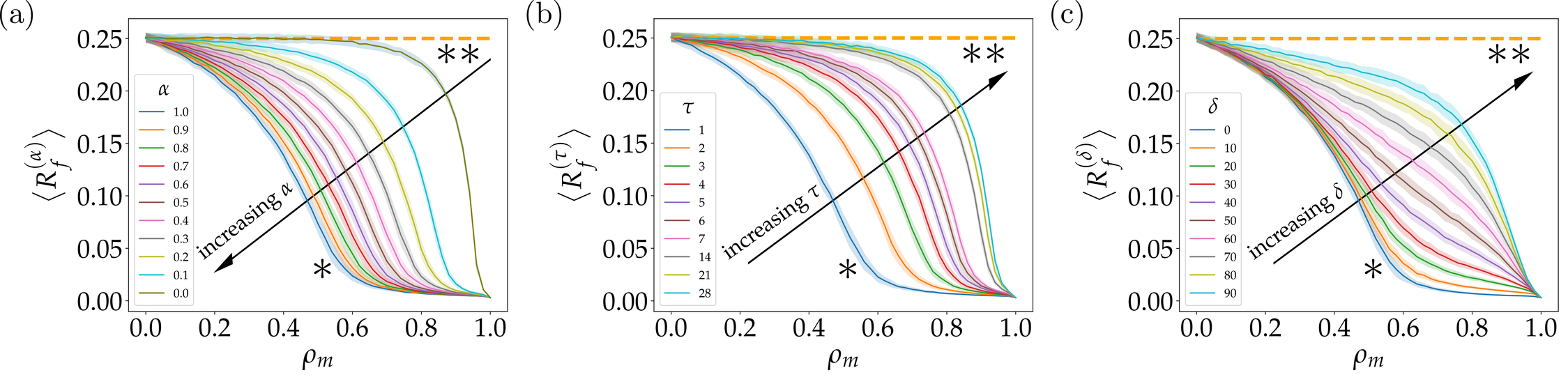

Supplement: S4 Fig — We replicate the analysis in Fig 3 but instead of susceptible agents now we have removed agents. All panels show the final ratio of removed agents after 1000 days of simulation for different values of ρm. (a) Central information delivered to the population while the ratio of informed agents changes. Different curves represent different ratios of informed agents α, ranging from 0 (olive line) to 1 (blue line) with 0.1 interval. (b) Periodicity of information delivery. Different curves represent different periodicity of information τ, delivered to the population. We have tested periodicity daily from 1 day (blue line) to 7 days (pink line), and then weekly, at 14, 21 and 28 days (green line). (c) Delay in starting information delivery. Different curves represent different delays δ, considering the elapsed time to deliver the message from the beginning of the epidemic. We have tested delay in the first message from 0 (blue line) to 90 days (light blue line) with 10 days interval. After the first message arrives, subsequent information is delivered daily. In all panels, shaded areas represent the standard deviation for 100 simulations. A * under blue line indicates the ideal strategy, and ** below orange dashed line indicates the worst strategy, i.e., when there is no central information delivered to the population neither information delivered to infected agents. Curves with lower values of 〈Rf(x)〉 where x ∈ {α, τ, δ} implied a better strategy result, i.e., less agents that were infected and then removed. (TIF) [file pone.0257995.s005.tif]

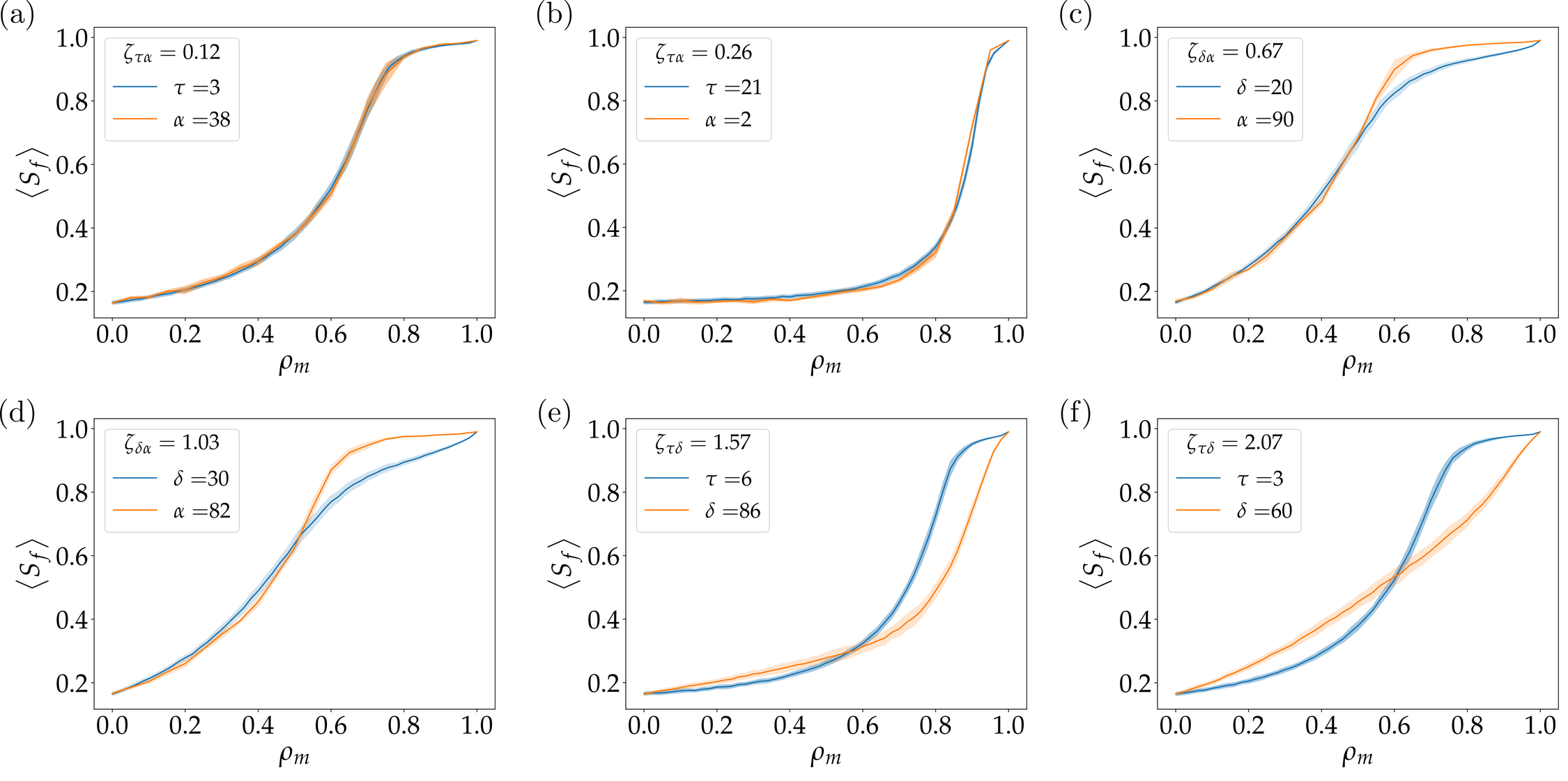

Supplement: S5 Fig — Here we show some examples of how similar is one strategy compared to another, where ζxy denotes the differences between curves x and y. (a) Comparison of informing each 3 days the whole population and the 38 percent daily. (b) Comparison of informing each 21 days the whole population and the 2 percent daily. (c) Comparison of informing with a delay of 20 days the whole population and to inform daily the 90 percent of population. (d) Comparison of informing with a delay of 30 days the whole population and to inform daily the 82 percent of population. (e) Comparison of inform each 6 days the whole population and with a delay of 90 days. (f) Comparison of inform each 3 days the whole population and with a delay of 60 days. (TIF) [file pone.0257995.s006.tif]
