# Supplementary material for: On the effectiveness of communication strategies as non-pharmaceutical interventions to tackle epidemics
Source: PLoS One. 2021 Oct 29;16(10):e0257995. doi: 10.1371/journal.pone.0257995 (PMC8555801; doi:10.1371/journal.pone.0257995)
Supplement: S1 Table — This values are for 2014–2016 Ebola outbreak in West Africa, being TE time that spend a person in Exposed state before become Infected, TI time that spend a person in Infected state before Dead or Recover, TD time that spend a person in Dead state before it get buried, f fraction of infected individual that die, βI the infection rate of Infected to Susceptible person and βD the infection rate of Dead to Susceptible person. These parameters were extracted from [36]. (PDF) [file pone.0257995.s007.pdf]

On the effectiveness of communication strategies as non-pharmaceutical interventions to tackle epidemics

Alejandro Bernardin, Alejandro J. Martínez, Tomas Perez-Acle

Parameters SEIRD model

Parameter	T_E	T_I	T_D	f	β_I	β_D
Value	11	6	4	0.7	0.25	0.20
