# Supplementary material for: On the effectiveness of communication strategies as non-pharmaceutical interventions to tackle epidemics
Source: PLoS One. 2021 Oct 29;16(10):e0257995. doi: 10.1371/journal.pone.0257995 (PMC8555801; doi:10.1371/journal.pone.0257995)
Supplement: Attachment — Submitted filename: Plos one 2021-04-10 comments.pdf [file pone.0257995.s008.pdf]

Comments for PONE-D-21-09046

This paper evaluates the effectiveness of social and communication factors within the population during an epidemic outbreak. The authors use an agent-based susceptible-exposed-infected-recovered-dead (SEIRD), epidemiological model, focusing on simulation results to reach a series of conclusions about the effectiveness of population communication. The model is meaningful and the derivation of the main results sounds right. But it needs to be revised before the second-round consideration. Here are some comments for this manuscript.

1. The summary says, “Moreover, having a scheme of delivering daily messages makes a stark different in most cases compare to any other type of strategies”. I suggest that the author express the differences mentioned here.
2. This paper focuses on the impact of communication strategies on the outcome of the epidemic, so I believe that the simulation results should compare the dynamic changes of the five types of nodes in the system with or without a certain communication strategy, and only focusing on “S_f” may not be enough to illustrate the effectiveness of the strategy.
3. Page 8, the author introduces the simulation results of the three non-drug treatment strategies respectively and writes in the abstract that the second strategy is different from the other strategies. However, the paper does not compare and explain the differences in the results caused by the three strategies. Therefore, I recommend that the authors discuss the differences in the conclusions reached under the three strategies.
4. The manuscript has cited more than 70 references, many quotes are necessary, such as: page 1, line 6, 2-10; page 2, line 15,14-30; etc.
5. The explanation of Equation (1) on Page 6 is not clear enough.
6. Page 6, what’s the basis for setting the initial condition “$q_i(0)=100$”? Please explain.
7. Page 7, for homogeneous and heterogeneous societies, the author shows different distribution patterns through Equation (2) and Equation (3). What is the basis for doing so?
8. What is the reason for this phenomenon “ for example, we can jump from $S_f = 0.34$, for $\alpha = 0.3$,to $S_f = 0.51$,for $\alpha = 0.6$, or to $S_f = 0.34$, for $\alpha = 0.9$” in the first paragraph on page 10? Besides, the description of specific curves in the figure is not clear enough. I recommend the author add the legend to all elements in the simulation diagram for reading.
9. There is some voice, grammar, and other problems in the paper, which are suggested to be modified, for example,
   (1) line 194, page 5, “Let us say, we wanted to apply this model to describe the evolution of an epidemic in a certain society, we state that…”. There are problems with tense inconsistencies and punctuation, please check.
   (2) Line 171, page 5, “information state ρ_i” should be “information state r_i”, please check.
   (3) Line 287, page 8, “in case B’”” should be “in case B”, please check punctuations.
   (4) Some picture numbers don't match the text description. Such as “Fig 3d”, line 288, and “Fig 3e”, line 296, page 8. Please check.
