# Supplementary material for: On the effectiveness of communication strategies as non-pharmaceutical interventions to tackle epidemics
Source: PLoS One. 2021 Oct 29;16(10):e0257995. doi: 10.1371/journal.pone.0257995 (PMC8555801; doi:10.1371/journal.pone.0257995)
Supplement: Attachment — Submitted filename: review.pdf [file pone.0257995.s009.pdf]

The manuscript investigates a susceptible-exposed-infected-recovered-dead (SEIRD) model in homogeneous and heterogeneous populations combining epidemic spreading dynamics and information awareness. Different scenarios are investigated, with information coming from local interactions of agents, or from a central entity. One of the conclusions is that communication is a key to tackle epidemics, increasing the final number of susceptible individuals.

The paper is well written and the introduction gives a very nice contextualization of the problem, starting with the COVID-19 situation and the current literature about epidemic spreading, its relationship with human behavior, and the need for data. The proposed model is based on the approach by Funk et al (2009) modeling the "awareness" to parametrize the susceptibility to infection. In this work, the authors investigate different strategies with an agent-based model framework in which agents can be informed with agent-agent interactions, or from a central source to reduce their infection susceptibility. The model has some limitations, such as the assumption that all information is trustworthy.

To introduce the model, the authors discuss an application on the 2014-2016 Ebola outbreak in East Africa. Indeed, it is a very good example of the importance of communication to change human behavior when highly effective pharmaceutical interventions are not available. The authors compare different strategies and their equivalence in outcomes using parameters for Ebola. I have some concerns about specific details of the model and its results, although I believe they are correct according to the construction of the model.

Major points:

- lines 135-151: What is the initial position of agents? Uniformly distributed, and then following a 2D random walk in a 2D lattice with periodic conditions? Or do they start from similar positions?
- lines 136,137,170,171,184,188,189: At the beginning, the "information state" is referred to as r_i. Later, ρ_i appears with the same name. In line 184 r_i appears again as information state, and in lines 188-189 ρ_i is defined as "decay constant". It was quite confusing to understand what is the relationship between r_i, ρ_i and q_i at first.
- lines 150-151 and Fig S1: What are the ODE equations that were compared with the ABM?
- line 212: The maximum quality of information happens with $q_i = 0$. I can understand $q_i = 1$ when the agent is exposed to a global information, and $q_i(t+1) = q_i(t) + 1$ in other cases (quality decays over time). However, when agents i and j interact, is there any particular reason to sum 2 to the highest quality among i and j?
- lines 225-232: Traditional ODE-based compartmental models assume a well-mixed population, but it is possible to build ODE equations considering heterogeneous mixing (see heterogeneous and quenched mean-field theories on complex networks, for example). It can be harder to write the equations and have the equivalence with ABMs, but it is not impossible.
- line 252 and captions of Fig 2 and 4: For the truncated Gaussian distribution, ρ_m is defined as the mode of the distribution. However, in the caption of Fig 2, it is referred to as the median. The same happens in Fig 4.
- lines 240-,303-307: The heterogeneity in the population is imposed by the decay constant distributions $p_1(\rho)$ and $p_2(\rho)$. When not truncated, the Gaussian is a homogeneous distribution (no heavy tails are present). Do the conclusions in lines 303-307 hold if other

heavy-tailed distributions are tested, such as a Cauchy or other power-law distributions? I am not sure if the truncated Gaussian alone is enough to conclude this.

- lines 240-,303-307: If the current results are for ρ_m as the mode, would not it be better to compare $p_{1,2}(\rho)$ with the same average?
- lines 356-358: "Opposite" in what sense? "then the results are opposite". I guess it is not the right word: the difference of S_f is smaller, but not "opposite".
- line 369 (Strategy II): I suggest to include a discussion on the difference between the time scale of the spreading dynamics and of the information spreading (with τ)
- line 369 (Strategy II): How to contextualize these results with the ones of Phys. Rev. Research 2, 023181 (2020) [DOI:10.1103/PhysRevResearch.2.023181], in which they investigate the effect of pulsating campaigns?
- lines 414-416: The curves delays are related to δ, not τ, right? "Now, instead of comparing curves of delays τ, we compare different τ for the same societies".
- line 482-: In this section, only communication between people is activated. It was not clear to me if the communication still happens when agents become infected, with $q_i(t+1) = 1$. This information is important to understand the results. If $q_i(t)$ is always increasing, it is expected that the outcome of this strategy is not effective.
- general question 1: How to connect these results with social media? It is not a "centralized" entity but has huge importance in communication and can be an alternative when the campaigns from the central government are absent.
- general question 2: What would happen to the results if a transition from R to S exists? In the case of COVID-19, for example, reinfection is possible, especially with the new variants.

Minor points:

- Fig S1: Why not use the ratio instead of percentage in the legend? If using percentage, it would be better to add an "%" sign.
- Fig 2(d), 2(e), and 4(c) should have a label on the color bar.
- line 287: Should be B' (not two primes)
- lines 288,296: The reference should be Fig 2, not 3.
- caption of Fig 3: It shows 1000 days of simulation, but the main text says 600 days
- line 347: Is there any statistical analysis to conclude the similarity with a "Chapman-Richard function"?
- lines 404-: If I am not mistaken, here the "difference" is between the curves for S_f, correct? That could be more clear.
