# Supplementary material for: On the effectiveness of communication strategies as non-pharmaceutical interventions to tackle epidemics
Source: PLoS One. 2021 Oct 29;16(10):e0257995. doi: 10.1371/journal.pone.0257995 (PMC8555801; doi:10.1371/journal.pone.0257995)
Supplement: Attachment — Submitted filename: Response to Reviewers.pdf [file pone.0257995.s010.pdf]

Fundación
Ciencia
& Vida

June 28, 2021

Fundación Ciencia y Vida
Computational Biology Lab
1482 Zañartu
Santiago, Chile
Phone: (+562) 2367-2000
Email: martinez@dlab.cl

Dear Dr. Gonçalves,

By this letter, we are submitting our responses to the comments we received for our manuscript number PONE-D-21-09046 entitled "On the effectiveness of communication strategies as non-pharmaceutical interventions to tackle epidemics". As you will see in the following paragraphs, we have made a thorough effort to address all the issues raised by the reviewers. In doing so, we expect that now our article could fit the standards needed for publication in your prestigious journal. We appreciate this opportunity to thank you and to the reviewers for taking the time to consider our work for publication.

Sincerely yours,

Alejandro J. Martínez (on behalf of all authors)
Dphil in Mathematics
Researcher at the Computational Biology Lab
Fundación Ciencia y Vida

Reviewer 1

1. **Comment:**
   The summary says, "Moreover, having a scheme of delivering daily messages makes a stark different in most cases compare to any other type of strategies". I suggest that the author express the differences mentioned here.
   Response:
   We would like to thank the reviewer for pointing us towards this issue. We wanted to refer to the fact that sending daily messages resulted in the best outcome between all the evaluated strategies. Also, we have a typographical error: *different* should be *difference*. Now we have corrected the abstract to be read: "Moreover, having a scheme of delivering daily messages makes a stark difference on the reduction of cases, compared to the other evaluated strategies, denoting that daily delivery of information produces the largest decrease in the number of cases."

2. **Comment:**
   This paper focuses on the impact of communication strategies on the outcome of the epidemic, so I believe that the simulation results should compare the dynamic changes of the five types of nodes in the system with or without a certain communication strategy, and only focusing on "Sf" may not be enough to illustrate the effectiveness of the strategy.
   Response:
   This is an interesting issue raised by the reviewer. For completeness, we have added the simulation results for cumulative dead, and removed agents. It is worth noting that agents at the end of the simulation can be in three possible states: susceptible, removed, and dead. This information allows us to evaluate the final impact of the epidemic. Since the exposed and infected agents are both transient, becoming zero at the end of the simulation, we have avoided them. We have added this complementary analysis in "Supporting information", S3 Fig and S4 Fig. This is mentioned on page 20, line 510.

3. **Comment:**
   Page 8, the author introduces the simulation results of the three non-drug treatment strategies respectively and writes in the abstract that the second strategy is different from the other strategies. However, the paper does not compare and explain the differences in the results caused by the three strategies. Therefore, I recommend that the authors discuss the differences in the conclusions reached under the three strategies.
   Response:
   We have an entire section called "Accessing the similarities between strategies" where we compare the applied strategies, describing when and how the evaluated strategies are equivalent. This section can be reviewed on page 20, line 515.

4. **Comment:**
   The manuscript has cited more than 70 references, many quotes are necessary, such as: page 1, line 6, 2-10; page 2, line 15,14-30; etc.
   Response:
   We have corrected the references, disaggregating many of them, as suggested by the reviewer. On the other hand, there are still some joint references, as is the case on page 2, line 14, where all of the cited work is about how media and information affect the spread of infectious diseases. Importantly, no textual references were made in the document.

5. **Comment:**
   The explanation of Equation (1) on Page 6 is not clear enough.
   Response:
   We thanks to the reviewer for rising this issue. Now we have expanded and ordered our explanation to make it clearer. We have changed the explanation starting on page 9, line 223. The new text is: "As stated before, new information can be gathered by agents through three different sources: i) through the action of a global source that periodically feeds information into the system, setting $q_i(t) = 0$ for all the informed agents; ii) from direct contact with agents in the same patch having information of higher quality, setting $q_i(t) = q_j(t) + 1$; or by iii) acquiring the disease, in which case the exposed agent gets informed when receiving the virus, setting $q_i(t) = 0$. Furthermore, information quality degrades in time, one unit per time iteration. Thus, for the case of $t + 1$, i.e. when a time unit goes by in the simulation, and according to the previous paragraph, the information quality constant of an agent i can turn into:

$$q_i(t+1) = \begin{cases} 1 & \text{if either agent } i \text{ is exposed to global information} \\ & \text{or agent } i \text{ is infected at time } t \\ q_j(t) + 2 & \text{if agents } i \text{ and } j \text{ interact and } q_j(t) < q_i(t) \\ q_i(t) + 1 & \text{otherwise} \end{cases} . \tag{1}$$

   Of note, when agents i and j interact, information transfer from an agent with higher quality to an agent with lower quality occurs, and simultaneously, a time unit goes by in the simulation. Hence, we add one unit because of communication and one unit because time goes by, resulting in $q_i(t + 1) = q_j(t) + 2$".

6. **Comment:**
   Page 6, what's the basis for setting the initial condition "qi(0)=100"? Please explain.
   Response:
   As denoted by the reviewer the explanation was missing in the text. Now the text, page 10, line 240, says: "Despite unbounded, we did set $q_i(0) = 100$ because it is a number representing low information quality such, when applied to $r_i = \rho^{q_i}$, it gives a result close to zero. In other words, when $q_i(0) = 100$, for $\rho \in [0.1, 0.9]$ the information state $r_i \in [10^{-100}, 2 \times 10^{-5}]$, meaning that agents have a negligible awareness of the pandemic situation."

7. **Comment:**
   Page 7, for homogeneous and heterogeneous societies, the author shows different distribution patterns through Equation (2) and Equation (3). What is the basis for doing so?
   Response:
   The basis for doing so is to have both a homogeneous and a heterogeneous distribution for the decay constant ρ. In doing so, we explored the impact of having different distributions of this parameter. To improve the clarity on this topic, we decided to change and expand our explanation. The resulting paragraph, on page 11, line 271, now is read: "More importantly, we explored the effect of having homogeneous and heterogeneous societies, by considering both the information quality q_i that each agent has in the system, and different distributions of the awareness decay constant $p(\rho)$. Of note, as q_i changes along simulation time following the interaction dynamics between agents, heterogeneity of information quality is expressed by the different distributions of q_i that may arise from the simulation. On the other hand, as the awareness decay constant ρ is defined as a parameter of the simulation, to enforce heterogeneity we sampled a distribution of ρ considering that $\rho_i \in [0, 1]$. Hence, two options arise: i) the first one representing a homogeneous society, where all agents have the same awareness decay constant $\rho_i = \rho_m$, where ρ_m represents the mode of the distribution. Therefore, the decay constant for such a society could be formally described as sampling ρ_i from a delta distribution, given by

$$p_1(\rho) = \delta(\rho - \rho_m)\,, \tag{2}$$

where δ is the Dirac delta and ρ_m represents its center. The second case corresponds to a ii) heterogeneous society, for which the decay constant can be obtained by sampling ρ_i from a truncated Gaussian distribution, given by

$$p_2(\rho) = \frac{\sqrt{2}}{\sigma\sqrt{\pi}} \frac{e^{-\frac{1}{2}\left(\frac{\rho-\rho_m}{\sigma}\right)^2} H(\rho)H(1-\rho)}{\text{erf}\left[\rho_m/\sqrt{2}\sigma\right] - \text{erf}\left[(\rho_m - 1)/\sqrt{2}\sigma\right]}\,. \tag{3}$$

In this case, σ is the standard deviation of the associated untruncated Gaussian distribution (which was chosen as $\sigma = 0.2$), ρ_m is the mode of the distribution, H is the Heaviside step function, and erf is the Gauss error function. Examples of truncated Gaussian distributions p_1 and p_2 using different values of ρ_m, are shown in panels (a) and (b) from Fig 2, respectively."

8. **Comment:**
What is the reason for this phenomenon "for example, we can jump from $S_f \approx 0.34$, for $\alpha = 0.3$, to $S_f \approx 0.51$, for $\alpha = 0.6$, or to $S_f \approx 0.69$, for $\alpha = 0.9$" in the first paragraph on page 10? Besides, the description of specific curves in the figure is not clear enough. I recommend the author add the legend to all elements in the simulation diagram for reading.
Response:
We thank the reviewer for rising this issue. It clearly deserves a deeper and better explanation. This phenomenon is due to the high non-linearity of the system, as can be read on page 17, line 410: "As noted in Fig 3a, there is an evident non-linear relationship between α and ρ_m. Particularly, we can see that our system behaves similarly for different values of α when ρ_m is close to 0.0 or close to 1.0, but for intermediate values of ρ_m, the high non-linearity of the system arise, denoted by a higher difference for the α curves. When ρ_m is close to 0.0, all the population have a fast decay of awareness, and inversely, when ρ_m is close to 1.0 all the population have a slow decay of awareness, so the percentage of informed people α does not contribute substantially to the final size of the epidemic. Now, for an intermediate case of ρ_m, there is not a clear predominance of the agents with fast decay of awareness or agents with slow decay of awareness, resulting in a substantial difference when informed different fraction of population α." We have also added legends to the figures, as suggested by the reviewer.

9. **Comment:**
   There is some voice, grammar, and other problems in the paper, which are suggested to be modified, for example,
   - *line 194, page 5, “Let us say, we wanted to apply this model to describe the evolution of an epidemic in a certain society, we state that. . . ”. There are problems with tense inconsistencies and punctuation, please check.*
   - *Line 171, page 5, ”information state ρ_i” should be ”information state r_i”, please check.*
   - *Line 287, page 8, “in case B”” should be “in case B”, please check punctuations.*
   - *Some picture numbers don’t match the text description. Such as “Fig 3d”, line 288, and “Fig 3e”, line 296, page 8. Please check.*

   Response:
   We apologize by these clumsy mistakes. They were fixed through the entire manuscript.

Reviewer 2

Major points:

1. **Comment:**
   lines 135-151: What is the initial position of agents? Uniformly distributed, and then following a 2D random walk in a 2D lattice with periodic conditions? Or do they start from similar positions?
   Response:
   The agents were uniformly distributed in the 2-torus space at the start of the simulation. A 2D lattice with periodic condition is a 2-torus, which is mentioned in the text. Anyway, we have added this explanation in the article for clarification, so now the text explains: ”Agents were uniformly distributed in the 2-torus space at the start of the simulation.” on page 7, line 157 and ”...we assumed that each agent moves in a 2-torus (2D space with periodic boundary conditions)” on page 7, line 148.

2. **Comment:**
   lines 136,137,170,171,184,188,189: At the beginning, the ”information state” is referred to as r_i. Later, ρ_i appears with the same name. In line 184 r_i appears again as information state, and in lines 188-189 ρ_i is defined as ”decay constant”. It was quite confusing to understand what is the relationship between r_i, ρ_i and q_i at first.
   Response:
   We apologize for the sloppy typographical errors. Parameter ρ corresponds to the decay constant and r_i to the information state.

3. **Comment:**
   lines 150-151 and Fig S1: What are the ODE equations that were compared with the ABM?
   Response:
   We have added the equations in the Supporting Information material. We have also added the corresponding citation for the work where the model was originally presented, and improved the main text for clarity. Now, on page 7, line 162, the new text is: ”Thus, the number of total patches was selected to define a rate of interactions so to adjust our ABM to the results of the SEIRD model based on ODEs proposed to explain the EVD dynamics by Weitz and Dushoff [36]. For a deeper dive into the SEIRD model, see S1 Eq. The comparison between the results of our ABM model and the ODE model is shown in S1 Fig”

4. **Comment:**
   line 212: The maximum quality of information happens with $q_i = 0$. I can understand $q_i = 1$ when the agent is exposed to a global information, and $q_i(t+1) = q_i(t)+1$ in other cases (quality decays over time). However, when agents i and j interact, is there any particular reason to sum 2 to the highest quality among i and j?
   Response:
   We thank the reviewer by noting this issue. The original text was confusing regarding this point, and yes, there is a reason to sum 2 to the agent that receives the information. We sum one unit when information is transferred from an agent with higher quality to an agent with lower quality, and we also sum one unit as one day goes by. As both processes occur simultaneously, we add 2 to the equation. We have improved our explanation in the manuscript for a better understanding. Now, in the text we have explained: ”Of note, when agents i and j interact, information transfer from an agent with higher quality to an agent with lower quality occurs, and simultaneously, a time unit goes by in the simulation. Hence, we add one unit because of communication and one unit because time goes by, resulting in $q_i(t+1) = q_j(t) + 2$.”, which can be found on page 9, line 232.

5. **Comment:**
lines 225-232: Traditional ODE-based compartmental models assume a well-mixed population, but it is possible to build ODE equations considering heterogeneous mixing (see heterogeneous and quenched mean-field theories on complex networks, for example). It can be harder to write the equations and have the equivalence with ABMs, but it is not impossible.
Response:
We thank the reviewer for pointing out overlooked methods. We have added the suggested information in the manuscript. We have added on page 11, line 257, the following: ”Heterogeneous and quenched mean-field theories using representations of the space-based on complex networks [72], can also be used to represent heterogeneity in dynamical systems. However, ABM models represent a straightforward way to deal with heterogeneity, since specific features that are unique to each agent can be individually associated.”.

6. **Comment:**
line 252 and captions of Fig 2 and 4: For the truncated Gaussian distribution, ρ_m is defined as the mode of the distribution. However, in the caption of Fig 2, it is referred to as the median. The same happens in Fig 4.
Response:
We thank to the by rising this notation error. ρ_m is indeed the mode of the distribution. We have fixed this.

7. **Comment:**
lines 240-,303-307: The heterogeneity in the population is imposed by the decay constant distributions $p_1(\rho)$ and $p_2(\rho)$. When not truncated, the Gaussian is a homogeneous distribution (no heavy tails are present). Do the conclusions in lines 303-307 hold if other heavy-tailed distributions are tested, such as a Cauchy or other power-law distributions? I am not sure if the truncated Gaussian alone is enough to conclude this.
Response:
We thank the reviewer for this interesting question. Our goal was to obtain a heterogeneous distribution centered in some specific value of ρ, to then compare the resulting simulations with the results of a simulation with a delta distribution centered in that same ρ. In this way, we investigated up to what extent our homogeneous system is similar to that of the heterogeneous distribution. Certainly, we could try different distributions to evaluate the equivalence between systems, but our objective is to show that, at least with a slight heterogeneity, the systems are equivalent. With a slight heterogeneity, we refer to a distribution where not heavy tails are present. Because of this, to compare a delta distribution against a truncated Gaussian distribution is enough to meet our requirements. We have greatly improved subsection ”On the influence of homogeneity and heterogeneity of agents”, to better convey this idea and also explicitly written on page 14, line 347, as follows: ”It is worth noting that if

Fundación Ciencia & Vida

we could certainly test many distributions to investigate the equivalence of the system, a truncated Gaussian distribution is enough to prove that the system behaves similarly with a slight degree of heterogeneity. With a slight degree of heterogeneity, we refer to a distribution where not heavy tails are present."

8. **Comment:**
lines 240-,303-307: If the current results are for ρ_m as the mode, would not it be better to compare $p_{1,2}(\rho)$ with the same average?
Response:
We think that the mode is a better descriptor for a truncated Gaussian distribution, this is because, in a unimodal distribution, the mode represents a central representative value but with some distribution around it. We have added the explanation on page 12, line 290, as follow: "Importantly, we decided to use the mode of the truncated Gaussian distribution so to produce a distribution of ρ surrounding a central representative value ρ_m, which can be understood as a delta distribution with lateral non-symmetrical diffusion, in both directions. As noted in Fig 2b, when producing a truncated Gaussian distribution of ρ using as center the ρ_m obtained from the Dirac delta (Fig 2a), three heterogeneous distributions of ρ, denoted A, B and C, were produced. Whereas distribution A resembles a skewed right distribution, distribution C resembles a skewed left distribution."

9. **Comment:**
lines 356-358: "Opposite" in what sense? "then the results are opposite". I guess it is not the right word: the difference of S_f is smaller, but not "opposite".
Response:
In one case the strategy is very effective, in the other, very inefficient, because of that we say "opposite", but we agree with the reviewer that it is not the best word, because of that we have changed the redaction for a better understanding. Now, on page 16, line 404, we have: "Nonetheless, if we perform the same analysis but this time we consider $\rho_m = 0.2$, the impact of the strategy is very limited, even if we have $\alpha = 1$, which is the ideal case.".

10. **Comment:**
line 369 (Strategy II): I suggest to include a discussion on the difference between the time scale of the spreading dynamics and of the information spreading (with τ)
Response:
We have included the discussion suggested by the reviewer and also added a new figure for a better explanation. Now, on page 18, line 446, the text is: ”To elaborate from another point of view on this conclusion, we include S2 Fig, where the final number of susceptible agents changes when the timescale of information, represented by τ, varies. Indeed, we can see in S2 Fig a fast decay for the firsts τ, and then a slow decay, representing a kind of ”long-tailed” decay, which denotes the importance of sending information with low periodicity — *i.e.* high frequency.”.

11. **Comment:**
line 369 (Strategy II): How to contextualize these results with the ones of Phys. Rev. Research 2, 023181 (2020) [DOI:10.1103/PhysRevResearch.2.023181], in which they investigate the effect of pulsating campaigns?
Response:
We thank the reviewer for presenting this interesting article. Similar to our strategy II, the authors study the effect of pulsating campaigns, and compare it to a continuous campaign. We have added the following discussion on page 18, line 452: ”In literature, we find a similar work [74], that also focuses on the effects of sending periodic information to the population, denoted by authors as ”pulsating campaigns”, under the assumption that there is a communication of the *risk* towards people, which fulfills a role similar to awareness in our work. Their conclusion regarding this type of campaign is that it is better a pulsating campaign instead of a campaign where the people are informed constantly, all this under an oscillatory dynamics of infection, *i.e.* where the infected cases grow, decay, and then repeat this dynamic. They explain this result as a consequence of an abrupt increase in risk communication when starting a campaign. Despite is not possible a direct comparison, as we don’t have oscillatory dynamics of infection, we can make some assumptions, for instance, that if we had oscillatory infection dynamics, we probably wouldn’t have some abruptly increase of awareness (doing a simile with risk definition), and due to this, contrary to [74], it is probable that a continuous strategy would be better in our model (shorter periods are better for the population in our model). Without a doubt, delving into these types of strategies can show us optimal ways to reduce infections.”

12. **Comment:**
lines 414-416: The curves delays are related to δ, not τ, right? "Now, instead of comparing curves of delays τ, we compare different τ for the same societies".
Response:
Yes, the curves are related to δ. We have fixed this, and now the text on page 19, line 492 reads: "Now, instead of comparing curves of delays δ, we compare different δ for the same society."

13. **Comment:**
line 482-: In this section, only communication between people is activated. It was not clear to me if the communication still happens when agents become infected, with $q_i(t+1) = 1$. This information is important to understand the results. If $q_i(t)$ is always increasing, it is expected that the outcome of this strategy is not effective.
Response:
In this section, people who have been infected do not receive new information, and as the reviewer points out, $q_i(t)$ is always increasing. As the reviewer comments, the result of this strategy is expected to be ineffective, but it is not expected to have zero impact, even when the entire population is informed. We have clarified both points in the manuscript, the first one as: "First, we explore the impact on the epidemic when there is only communication between agents, without central information delivery. In other words, agents do not receive information from mass media/central entity, neither infected agents receive information when acquiring the virus. Therefore, the only source of information is the initial information that agents have at the beginning of the simulation.." on page 22, line 568. The second one has been modified as "Although is expected that this strategy is not effective, due to the decay of information quality in time, given by $q_i(t + 1) = q_i(t) + 1$, what it is surprising is that it has zero impact even when all population is informed at the beginning of the simulation.", on page 23, line 581.

14. **Comment:**
general question 1: How to connect these results with social media? It is not a "centralized" entity but has huge importance in communication and can be an alternative when the campaigns from the central government are absent.
Response:
We thank the reviewer for this interesting question. Social media could be considered at the intersection between a central entity and people communication. Similar to central entity communication, it also has a huge impact and could reach a lot of people, and compared with people communication, it depends on agent connection network. It also has the characteristic that it is a media where we can find unreliable information, also knows as "fake news", which could lead to an interesting different dynamics, compared with a system where there is only trustworthy information. Despite this is an interesting topic, it is out of the scope of the present paper, but without any doubt, we will consider it for future work.

15. **Comment:**
general question 2: What would happen to the results if a transition from R to S exists? In the case of COVID-19, for example, reinfection is possible, especially with the new variants.
Response:
This is also a very interesting question, and we thank the reviewer for the comments. Despite what we can speculate, that the information strategies would decrease their effectiveness because the epidemic will last longer, and also that the system will show an oscillatory behavior, and probably the information received in certain infection wave could help to decrease the size of the next infection wave, this question is out of the scope of our work. Definitely, due to many interesting questions arising from this suggestion, we will consider it for future work.

Minor points:

- **Comment:**
Fig S1: Why not use the ratio instead of percentage in the legend? If using percentage, it would be better to add an "%" sign.
Response:
This was a mistake. Now, we have used ratio, as the reviewer suggests.
- **Comment:**
Fig 2(d), 2(e), and 4(c) should have a label on the color bar.
Response:
We have added labels to color bars.
- **Comment:**
line 287: Should be B' (not two primes)
Response:
This was a typographical error. We have fixed it.
- **Comment:**
lines 288,296: The reference should be Fig 2, not 3.
Response:
The reviewer is right. We have fixed this mistake.
- **Comment:**
caption of Fig 3: It shows 1000 days of simulation, but the main text says 600 days
Response:
It is 1000 days, it has been corrected. Around 600 days the system reaches an equilibrium state. Because of this we also have 2 plots that end in $t = 600$. We have also added this explanation on page 15, line 366: "As we previously proceeded, in all three strategies we are interested in the outcome of the epidemic by evaluating the number of susceptible agents at the end of the simulation, *i.e.* at $t = 1000$ [days]. However, at $t = 600$ [days] the system has virtually reached the equilibrium state"

- **Comment:**
  line 347: Is there any statistical analysis to conclude the similarity with a "Chapman-Richard function"?
  Response:
  No, there is no statistical analysis to conclude this, this was a qualitative observation. We have decided to erase this from text, to avoid misunderstanding.

- **Comment:**
  lines 404-: If I am not mistaken, here the "difference" is between the curves for S_f, correct? That could be more clear.
  Response:
  Yes, the difference is between curves for S_f, but we are comparing different societies, which are represented by different ρ_i. We have improved the explanation to avoid this misunderstanding. Now the text on page 19, line 483 is: "This effect becomes even clearer when comparing three different societies, *i.e.* three different ρ_m, through its different curves S_f.
