# Supplementary material for: On the effectiveness of communication strategies as non-pharmaceutical interventions to tackle epidemics
Source: PLoS One. 2021 Oct 29;16(10):e0257995. doi: 10.1371/journal.pone.0257995 (PMC8555801; doi:10.1371/journal.pone.0257995)
Supplement: Attachment — Submitted filename: Plos one 2021-07-22.pdf [file pone.0257995.s011.pdf]

The author used an epidemiological model of SEIRD based on the agent approach, and answered and improved the questions raised previously. However, we still have some questions as follows.

Comment 1. On page 5 of the article, line 77, the author mentions "Even though we are in the middle of a COVID-19 pandemic, and considering the worldwide context, it would have made more sense to work in those lines, our understanding about EVD is fare more mature than that of COVID-19". So the article is based on the EVD virus to verify. However, the EVD virus and COVID-19 are significantly different in the incubation period and even fatality rate. How to prove the universality of the model?

Comment 2. The picture with the legend provided by the author is not clear enough. At the same time, the author mentioned using the actual data of the Ebola virus for simulation before, but the article did not introduce how to deal with the data, so the conclusion obtained seems not credible.

Comment 3. The evaluation of the effectiveness of information transmission in this paper can provide methods and strategies for the prevention of infectious diseases. However, the transition between the introduction and conclusion is relatively abrupt and inconsistent, so we suggest improving it.

Comment 4. page 8, line 185, We note that you mentioned: "$r_i = 0$ indicates that the agent is completely unaware of the epidemics". As for the form of information state function you set, with the time iteration in the system, the information quality will gradually improve, while the individuals in the system have a declining understanding of infectious diseases. We are puzzled about this, and hope the author can further explain it.

Comment 5. This paper studies the influence of communication strategy based on the epidemic model, SEIRD. For this model, whether the system eventually approaches the

disease-free equilibrium, namely $e = 0, i = 0$, depends largely on its own threshold condition. We suggest that the communication strategy should be further explained in the light of the situation that the system tends to the endemic equilibrium.

Comment 6. In sup Figure1, the final result of the SEIRD model under the ABM framework in (a) is not much different from that in Figure (b). Is it insufficient to support the conclusion that the communication strategy can effectively control the spread of the disease?
