# Supplementary material for: On the effectiveness of communication strategies as non-pharmaceutical interventions to tackle epidemics
Source: PLoS One. 2021 Oct 29;16(10):e0257995. doi: 10.1371/journal.pone.0257995 (PMC8555801; doi:10.1371/journal.pone.0257995)
Supplement: Attachment — Submitted filename: Response to Reviewers.pdf [file pone.0257995.s012.pdf]

Fundación Ciencia & Vida

September 8, 2021

Fundación Ciencia y Vida
Computational Biology Lab
1482 Zañartu
Santiago, Chile
Phone: (+562) 2367-2000
Email: martinez@dlab.cl

Dear Dr. Gonçalves,

By this letter, we are submitting our responses to the comments of the second round of review that we received for our manuscript number PONE-D-21-09046 entitled "On the effectiveness of communication strategies as non-pharmaceutical interventions to tackle epidemics". As you will see in the following paragraphs, we have answers the six questions rised by Reviewer 1, including the one referring to Supplementary Figure 1. In doing so, we expect that now our article could fit the standards needed for publication in your prestigious journal. We appreciate this opportunity to thank you and to the reviewers for taking the time to consider our work for publication.

Sincerely yours,

Alejandro J. Martínez (on behalf of all authors)
Dphil in Mathematics
Researcher at the Computational Biology Lab
Fundación Ciencia y Vida

Reviewer 1

1. **Comment:**
   On page 5 of the article, line 77, the author mentions "Even though we are in the middle of a COVID-19 pandemic, and considering the worldwide context, it would have made more sense to work in those lines, our understanding about EVD is fare more mature than that of COVID-19". So the article is based on the EVD virus to verify. However, the EVD virus and COVID-19 are significantly different in the incubation period and even fatality rate. How to prove the universality of the model?
   Response:
   As mentioned by the reviewer, our article is based on the Ebola virus disease (EVD), which indeed behaves significantly different from COVID-19. However, from a modeling perspective, they both share similar spreading principles that can be captured using compartmental models. This is important because, even though we presented results that integrate the information phenomenon with the SEIRD model, this integration could have been accomplished using any other compartmental model, in this sense our approach is somehow universal. Nevertheless, we have worked with the SEIRD model because it has shown to be very effective at describing the evolution of EVD in a population, and also the EVD parameters, such as infection rates, incubation period, among others, are well described in the literature. Regarding COVID-19, the literature has not reached enough maturity and there is still no consensus in the community on the models and parameters that best describe its behavior. For this reason we decided to stick with EVD, nonetheless working with COVID-19 could have been more sounding.
   Regarding universality, as mention before, our results are based on EVD, and therefore, they should not be treated as universal, in the sense that cannot be transferred to other diseases directly. For instance, if one wanted to apply our model to COVID-19, despite exhibiting a SEIRD-compatible dynamics, then the parameters should be set to the specific values of COVID-19. For clarity we have added a paragraph in the conclusion, please see page 25, line 633.

2. **Comment:**
   The picture with the legend provided by the author is not clear enough. At the same time, the author mentioned using the actual data of the Ebola virus for simulation before, but the article did not introduce how to deal with the data, so the conclusion obtained seems not credible.
   Response:
   We assume that the reviewer is referring to Figure 1. If this is the case, we are not convinced that this figure requires an amendment, because it was intended to be a graphical representation of both the SEIRD model and the ABM dynamics. Therefore, from our point of view, in its current form, Figure 1 achieves its goal.
   On the other hand, as we describe in methods, page 7, line 162, and page 8, line 183, we did not use real data to fit our model. On the contrary, we extracted the EVD parameters from the literature and we used them to fit our ABM model results, to that of the ODE model proposed by Weitz and Dushoff[1]. As seen in S1 Fig, by comparing the peak days and the values of the final states, we conclude that our ABM reproduces the dynamics of an ODE model for EVD, which is currently found in the literature. It is important to mention that our conclusions are not solely based on the parameters for the EVD extracted from the literature, but also from our implementation on ABM of the information model proposed by Funk et. al.[2]. By joining these models we highlight the importance of proper communication strategies, both accurate and daily, to tackle epidemic outbreaks.

3. **Comment:**
   The evaluation of the effectiveness of information transmission in this paper can provide methods and strategies for the prevention of infectious diseases. However, the transition between the introduction and conclusion is relatively abrupt and inconsistent, so we suggest improving it.
   Response:
   We thank to the reviewer by noting the inconsistency between the introduction and the conclusion. We made some changes to the conclusion, to make it more consistent with the introduction. The new version of the text can be read in page 25, line 655. Also, we added some minor changes, which are highlighted in the marked-up copy of our work.

4. **Comment:**
page 8, line 185, We note that you mentioned: "$r_i = 0$ indicates that the agent is completely unaware of the epidemics". As for the form of information state function you set, with the time iteration in the system, the information quality will gradually improve, while the individuals in the system have a declining understanding of infectious diseases. We are puzzled about this, and hope the author can further explain it.
Response:
Importantly, in our model, the information quality $q(t)$ will gradually decrease along time. The only way in which $q(t)$ increases is when the agents acquire new information from a central entity. As $r_i(t) = \rho_i^{q_i(t)}$, when $q(t) = 0$ the agents achieve the maximum state of information. Consistently, a higher value for $q(t)$ implies less information quality, resulting in r_i close to zero. To avoid any misunderstanding in this complex topic, we have improved the explanation as follows: "Whilst $r_i = 0$ indicates that agent i is completely unaware of the epidemic, when $r_i = 1$, agent i becomes completely aware of the epidemic and the state of its environment, knowing exactly how to prevent the infection. We consider r_i as a function of time given by $r_i(t) = \rho_i^{q_i(t)}$, where $\rho_i \in [0, 1]$ is the *awareness decay constant* of agent i. On the other hand, $q_i(t) \in \mathbb{N}$ is the *information quality constant* of agent i, which is also a function of time, being $q(t) = 0$ the maximum information quality.", in page 8, line 200.

5. **Comment:**
This paper studies the influence of communication strategy based on the epidemic model, SEIRD. For this model, whether the system eventually approaches the disease-free equilibrium, namely $e_i = 0$, $i = 0$, depends largely on its own threshold condition. We suggest that the communication strategy should be further explained in the light of the situation that the system tends to the endemic equilibrium.
Response:
We appreciate this very interesting suggestion made by the reviewer. However, we consider it out of the scope of the present work and we certainly will pursue it in a future study.

6. **Comment:**
In sup Figure1, the final result of the SEIRD model under the ABM framework in (a) is not much different from that in Figure (b). Is it insufficient to support the conclusion that the communication strategy can effectively control the spread of the disease?
Response:
In Sup Figure 1 we show two systems without information. We made a comparison between the ABM and the ODE systems to evaluate the consistency of our model with current literature. In other words, that we are capable to replicate with our ABM system the ODE dynamics extracted from literature. This ABM is the base system that later on is complemented with the information model. To avoid any misunderstanding we improved the caption as follows: "**ABM evaluation against the literature** (a) ABM, temporal evolution of the states of the system for 100 repetitions. The shaded area is the standard deviation. (b) Same model in deterministic ODEs system. In both panel, curves represent the temporal evolution of the states of the system. The legend for each states represent the final ratio of agents at the end of the simulation. Maximum infected in ABM, 577 persons in $t = 129$ [days], maximum infected in ODE, 542 persons, in $t = 135$ [days]. It is worth noting that these systems do not include the information model."

References

[1] Weitz JS, Dushoff J. Modeling post-death transmission of Ebola: challenges for inference and opportunities for control. Scientific reports. 2015;5:8751.

[2] Funk S, Gilad E, Watkins C, Jansen VA. The spread of awareness and its impact on epidemic outbreaks. Proceedings of the National Academy of Sciences. 2009;106(16):6872–6877.
